# Engineering the FK228 biosynthetic gene clusters for high-yield production

**DOI:** 10.3389/fmicb.2026.1933794

**Published:** 2026-10-15

**Authors:** Maoqin Wang, Xuexin Jia, Yunling Zhang, Junchi Ma, Youming Zhang, Jun Fu, Ruijuan Li

**Affiliations:** 1 State Key Laboratory of Microbial Technology, Shandong University, Qingdao, China; 2 NHC Key Lab of Reproduction Regulation, Shanghai Engineering Research Center of Reproductive Health Drugs and Devices, Shanghai Institute for Biomedical and Pharmaceutical Technologies, Shanghai, China

**Keywords:** FK228, biosynthetic gene clusters, *Burkholderia thailandensis* E264, promoter engineering, TdpR

## Abstract

FK228 (romidepsin) is a potent HDAC inhibitor; however, its clinical application is severely constrained by low natural yields and the genetic intractability of its native producer. Here, we leveraged the established *Burkholderia* chassis KOGC1 to develop a high-performance platform for FK228 production. Through systematic fermentation optimization, we addressed major production constraints including potential end-product toxicity and precursor supply. Specifically, the integration of XAD-16 resin adsorption together with the combined feeding of key amino acids (cysteine, valine, and threonine) elevated the titer to 743.5 mg/L. Subsequent refactoring of the biosynthetic gene cluster and promoter engineering further increased the titer to 1109.4 mg/L. Furthermore, the engineered strain exhibited remarkable genetic stability over five generations without antibiotic selection, which may help address a key barrier to future scale-up. Overall, this study establishes a highly productive laboratory-scale FK228 biosynthetic platform, providing a foundation for future process development of this important therapeutic agent.

## 1 Introduction

Cancer remains a major global burden, with incidence and morbidity rising steadily (Li et al., 2024). Epigenetic dysregulation, particularly histone acetylation and deacetylation mediated by histone acetyltransferases (HATs) and histone deacetylases (HDACs), is closely linked to tumorigenesis (Borcoman et al., 2021). By altering histone lysine acetylation, these enzymes modulate chromatin transcription and cancer-related gene expression, making HDACs promising therapeutic targets. HDAC inhibitors (HDACi) suppress HDAC activity, increasing acetylation, reactivating tumor suppressors, inhibiting proliferation, inducing differentiation, restoring normal phenotypes, and triggering apoptosis (Glaser, 2007; Parveen et al., 2023; Shi et al., 2024). Hence, HDACi has become a focus of cancer research.

FK228 (Romidepsin, Istodax) is a cyclic peptide natural product isolated from *Chromobacterium violaceum* No. 968 (Vandermolen et al., 2011), with its clinical formulation approved by the FDA for cutaneous and peripheral T-cell lymphomas (West and Johnstone, 2014). Due to its complex structure, total synthesis remains challenging, making microbial fermentation the preferred production route. The biosynthetic gene cluster (*R-tdp-dep* BGC) encodes a *trans*-AT PKS-NRPS assembly line: loading of cysteine, valine, dehydrothreonine, and malonyl-CoA followed by sequential condensation, macrocyclization, and oxidative modification to yield the bioactive depsipeptide (Passmore et al., 2026). Cysteine and valine are directly incorporated while dehydrothreonine arises from threonine via dehydration, and malonyl-CoA serves as the acyl donor for PKS extension (Cheng et al., 2007). Understanding this precursor dependence is critical, as rational supplementation of during fermentation can directly elevate metabolic flux toward FK228, providing a foundation for the precursor engineering strategies explored in this study.

However, industrial production of FK228 has long been hampered by two interrelated bottlenecks: limited strain accessibility and low fermentation yields. To address these, alternative microbial sources have been explored. The thailandepsins gene cluster (*tdp*) was identified in 2011, producing FK228 analogs including thailandepsin A with potent antitumor activity (Wang et al., 2011). Subsequent fermentation optimization enabled large-scale thailandepsin A production, informing FK228 process development (Liu et al., 2012). Heterologous expression of the complete FK228 BGC in *E. coli* BL21, however, gave yields comparable to the native strain (19 mg/L) (Wesener et al., 2011). Later, genome mining revealed a highly homologous BGC in *B. thailandensis* MSMB43 which after minor modificationn produced 168.5 mg/L of FK228 (Liu et al., 2018).

Despite these advances, productivity remained a critical bottleneck. In 2022, our group adopted a combinatorial biosynthesis strategy to construct a hybrid FK228 BGC. Deletion of the native *tdp* BGC from *B. thailandensis* E264 and integration of an *attB* site generated the chassis strain KOGC1, which produced 581 mg/L, the highest reported at the time (Gong et al., 2023). Nevertheless, this yield still fell short of industrial requirements, underscoring the need for further strain engineering and process optimization.

*B. thailandensis* E264, a Gram-negative soil and freshwater isolate from Thailand, is a prolific producer of bioactive natural products (Funston et al., 2016), including thailandepsins (Biggins et al., 2012; Wang et al., 2012) and various other secondary metabolites (Kunakom and Eustáquio, 2019). Genome mining with antiSMASH has revealed abundant BGCs, particularly numerous polyketide synthase (PKS) and non-ribosomal peptide synthetase (NRPS) clusters, underscoring its potential to supply precursors for heterologous expression. Additionally, its rapid growth and simple cultivation requirements make it a highly promising chassis for high-yield production (Xu et al., 2020).

In this study, we engineered a high-yielding strain for FK228 production that achieved a record titer of 1,109.4 mg/L. By integrating rational fermentation optimization-specifically in-situproduct sequestration and synergistic precursor feeding-with targeted promoter refactoring, we effectively alleviated end-product toxicity and flux limitations. Crucially, the heterologous BGC maintained structural integrity and high-level production over five generations without antibiotic selection, resolving a critical barrier to industrial scalability. This work establishes the most efficient FK228 production platform to date and provides a generalized framework for stabilizing large, unstable gene clusters, thereby facilitating the cost-effective manufacture of complex natural products.

## 2 Materials and methods

### 2.1 Strains, plasmids and reagents

The strains, plasmids, and oligonucleotides used or generated in this study are listed in Supplementary Tables S1, S2, S3. Oligonucleotides were synthesized by Sangon Biotech (Shanghai, China). Escherichia coli strains were cultured in LB medium (tryptone, 10 g/L; yeast extract, 5 g/L; NaCl, 1 g/L; agar for solid medium, 15 g/L) at 37 °C with appropriate antibiotics: kanamycin (15 μg/mL) and chloramphenicol (20 μg/mL). *E. coli* GBred-gyrA462 was used for linear-plus-circular homologous recombination (LCHR). *E. coli* WM3064 was employed as a conjugal donor. The chassis strain KOGC1 was constructed in our laboratory by deriving it from *B. thailandensis* E264 wild-type (WT). The entire native thailandepsins biosynthetic gene cluster (*tdp* BGC; Chromosome I; 2,653,679-2,692,255, spanning genes BTH_I2355 through BTH_I2368) was deleted and replaced with a 34-bp *attB* integration site recognized by the *Streptomyces* phage ΦC31 integrase system. No antibiotic resistance markers were retained in the final KOGC1 chromosome after counterselection. KOGC1 was routinely propagated in LB medium at 37 °C and used for fermentation in M9 medium (glucose 17.89 g/L, tryptone 34.98 g/L, K_2_HPO4·3H_2_O 20.44 g/L, KH_2_PO4 4.4 g/L, sodium citrate 0.01 g/L, pH 7.0) supplemented with kanamycin (300 μg/mL) (Kang et al., 2011).

### 2.2 Promoter engineering and reconstruction of the FK228 biosynthetic gene cluster

The RedEx strategy was employed for promoter engineering of the FK228 BGC (Song et al., 2020). To enhance transcriptional regulation, a library of seven promoters (P11, P16, P17, P33, P35, P44, and P46) was inserted upstream of the pathway-specific regulator gene *tdpR*. Briefly, plasmid p15A-cm-R-tdp-dep was electroporated into *E. coli* GBred-gyrA462 (Schematic diagram of the FK228 biosynthetic plasmid in Supplementary Figure S1). Tandem PCR was performed to generate *Kpn*I-*kmccdB*-Px-*Kpn*I fragments flanked by 50-bp homologous arms (HAs). Using Redαβ-mediated linear–circular homologous recombination (LCHR), the *km-ccdB*-Px cassette was integrated upstream of *tdpR*, yielding p15A-cm-km-ccdB-Px-tdpR-tdp-dep. Following *Kpn*I digestion to remove the *km-ccdB* selection-counterselection marker, the linearized vector (500 ng) was circularized using 0.2 U Assembly Mix (ABclonal) at 50 °C for 1 h. The reaction mixture was desalted and electroporated into *E. coli* GB2005. Positive clones carrying the desired promoter replacements were identified by *Pst*I restriction analysis.

For chromosomal integration, a km-int-*attP* fragment containing 50-bp HAs was amplified and assembled into the promoter-engineered plasmids via LCHR, generating the final expression vectors p15A-km-int-attP-Px-tdpR-tdp-dep (where Px denotes the respective promoter). These constructs were subsequently integrated into the chromosome of KOGC1 via ΦC31 integrase-mediated site-specific recombination at the genomic *attB* site. Correct integration and single-copy insertion of the *Px-tdpR-tdp-dep* BGCs were verified by colony PCR.

### 2.3 Transcriptional regulator engineering

The transcriptional regulators DepR were engineered using the RedEx strategy to fine-tune BGC expression. The resulting plasmids, p15A-km-int-*attP*-depR-tdp-dep, were integrated into the chromosome of the chassis strain KOGC1 via ΦC31-mediated site-specific recombination. This allowed systematic evaluation of their regulatory effects on FK228 production.

### 2.4 Integration of attachment sites into the chromosome of the chassis strain

The recombinant plasmid was introduced into *E. coli* WM3064 via electroporation and verified by restriction analysis. For conjugation, *B. thailandensis* KOGC1 and *E. coli* WM3064 harboring the target plasmid were separately streaked onto LB agar plates. KOGC1 was cultured on antibiotic-free LB agar, while WM3064 was grown on LB agar supplemented with diaminopimelic acid (DAP, 200 μg/mL) and kanamycin (15 μg/mL). Both plates were incubated overnight at 37 °C. A single colony of WM3064 was then scraped and evenly spread over a freshly prepared lawn of KOGC1 on an antibiotic-free LB agar plate. After co-incubation at 37 °C for 6 h to allow conjugation, the cells were harvested and washed with fresh LB broth. The pellet was resuspended in 100 μL of LB and streaked onto selective LB agar plates containing kanamycin (300 μg/mL). Putative transconjugants were screened after overnight incubation at 37 °C and confirmed by colony PCR.

### 2.5 Fermentation and analytical methods

Seed culture was performed in two stages. A glycerol stock of strain was initially streaked onto LB agar containing kanamycin (300 μg/mL) and incubated at 37 °C for 24 h. A single colony was subsequently inoculated into a 250 mL baffled flask containing 50 mL of LB medium supplemented with kanamycin and cultivated at 37 °C with shaking (180 rpm) for 16 h to obtain the secondary seed culture.

For fermentation, the secondary seed culture was inoculated at the optimized inoculum size of 1% (v/v) into 65 mL of M9 medium in a 250 mL flask. The M9 medium was pre-supplemented with the three key precursors for FK228 assembly-cysteine, valine, and threonine (the precursor to dehydrothreonine)-each at a final concentration of 20 mg/L. The cultures were incubated at 28 °C and 180 rpm for 12 h, at which point 4% (w/v) XAD-16 macroporous resin was added. Fermentation continued under the same conditions for a total of 60 h from the time of inoculation. Upon completion, the culture broth was centrifuged (8,000 rpm, 5 min), and the cell pellet along with the adsorbed resin was collected. The mixture was extracted with 50 mL of ethyl acetate via sonication (10 min) followed by agitation (200 rpm, 3 h). After filtration, the solvent was evaporated under reduced pressure, and the crude extract was dissolved in 1 mL of HPLC-grade methanol and filtered through a 0.22 μm membrane.

Metabolite analysis was conducted using an Ultimate 3,000 UHPLC system (Thermo Fisher Scientific) coupled to an Impact HD micrOTOF-Q III mass spectrometer (Bruker Daltonics) equipped with an ESI source. Separation was achieved on an Acclaim RSLC 120 C18 column (2.1 × 100 mm, 2.2 μm) at a flow rate of 0.3 mL/min. The mobile phase consisted of water with 0.1% formic acid (A) and acetonitrile with 0.1% formic acid (B) using a gradient: 0–3 min, 5% B; 3–19 min, 5–95% B; 19–22 min, 95% B; 23–25 min, 5% B. Mass spectra were acquired in centroid mode over *m/z* 100–1,500 in positive ionization mode with automatic MS/MS fragmentation.

### 2.6 Determination of Glucose Concentration

Glucose concentrations in the fermentation broth were quantified using an SBA-40D Biosensor Analyzer. Prior to analysis, the autosampler needle was flushed three times with more than 25 μL of 1 g/L glucose standard solution (this flushing step was identically repeated for all test samples; following the final measurement, the needle was rinsed three times with ddH_2_O). For calibration, exactly 25 μL of the 1 g/L glucose standard was aspirated, any residual liquid on the needle tip was carefully wiped off with filter paper, and the sample was rapidly injected into the analyzer. Measurements commenced once the instrument indicated successful calibration. For sample analysis, the fermentation broth was first filtered through a 0.22 μm membrane and diluted with ddH_2_O as needed. Exactly 25 μL of the diluted filtrate was rapidly injected into the analyzer, and the displayed value was recorded. The final glucose concentration was calculated as: Glucose content = Displayed value × Dilution factor.

### 2.7 Quantitative analysis of FK228

An authentic FK228 standard (purity ≥ 99%; [MedMol], Lot [S80251]) was used for calibration and identity confirmation. Standard solutions at concentrations of 100, 200, 400, 800 and 1000 mg/L were prepared in HPLC-grade methanol. Quantification was carried out by HPLC-MS under the chromatographic conditions described in Section 2.5. Peak areas of FK228 were extracted from the extracted ion chromatogram (EIC) of the theoretical [M+H]? ion. A calibration curve was constructed by plotting peak area against concentration, fitted to a linear regression equation y = 1864772.821x + 3278281724 with *R*^2^ ≥ 0.99.FK228 titers in fermentation extracts were calculated according to the calibration curve, with corrections for extraction solvent volume, sample dilution factor and final resuspension volume. To verify peak identity, retention time, accurate mass error ( ≤ 5 ppm) and MS/MS fragmentation spectra of sample peaks were matched against the authentic standard(Supplementary Figure S2).

## 3 Results

### 3.1 Optimization of fermentation conditions enhances FK228 production

To establish fermentation parameters on a genetically unperturbed background, we used KOGC1/R-tdp-dep (harboring an intact FK228 BGC) as the host, ensuring that titer improvements arose from environmental rather than genetic factors. Although prior thailandepsin production studies in *B. thailandensis* E264 informed our approach (Liu et al., 2012), direct extrapolation was invalid due to the engineered deletion and integration in KOGC1. We therefore performed a systematic re-optimization using a sequential single-factor design, which allowed clear dissection of individual factor contributions but could not capture synergistic interactions-a limitation that future multivariate methods should address.

FK228 accumulation was strictly growth-associated. In M9 medium, the titer increased significantly after 24 h, peaked at approximately 628 mg/L at 60 h, and then declined, which may be attributable to end-product inhibition or nutrient depletion (Figures 1A, B). A 60-h harvest time was thus fixed for all subsequent experiments. Late-logarithmic cells (16 h) were used as seeds, and inoculum sizes ranging from 0.5% to 5% (v/v) were evaluated; 0.5%, 1%, and 2% yielded comparable titers, whereas 5% reduced production, likely resulting from high cell density and premature metabolite accumulation. Hence, 1% inoculum was adopted (Figure 1C). To alleviate potential toxicity and feedback inhibition, XAD-16 macroporous resin was introduced at 12 h post-inoculation. Screening resin concentrations based on prior thailandepsin fermentation identified 4% (w/v) as optimal, tripling the titer to ~650 mg/L compared with the resin-free control (Figure 1D). We speculate that the beneficial effect of XAD-16 resin is associated with *in-situ* sequestration of extracellular FK228, which may alleviate end-product toxicity and feedback inhibition; however, direct evidence such as intracellular/extracellular FK228 quantification, adsorption kinetics or cell viability assays was not obtained in this study, and the underlying mechanism requires further investigation.

**Figure 1:**
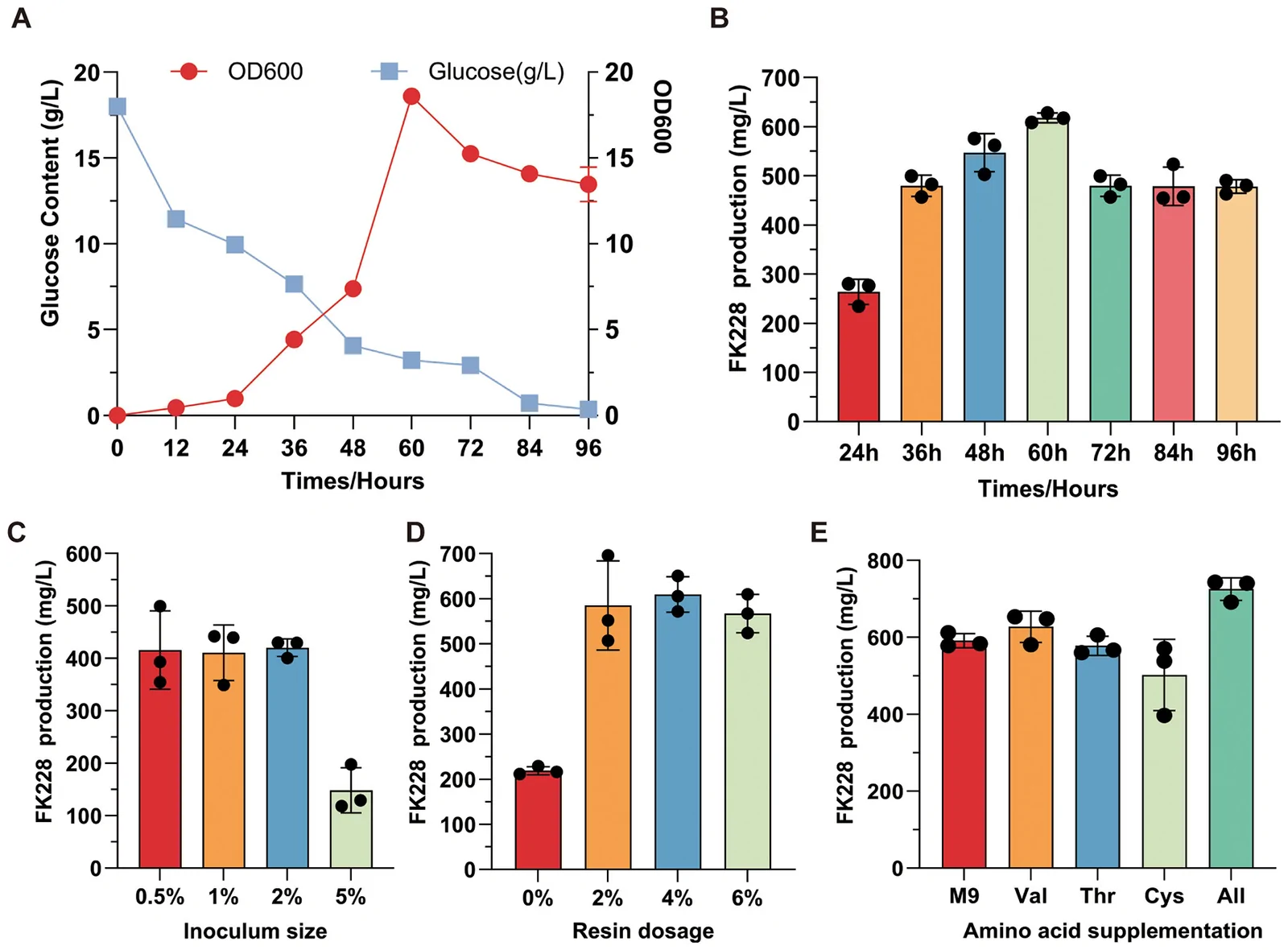
Preliminary optimization of fermentation conditions for *B. thailandensis* KOGC1 harboring the *R-tdp-dep* BGC. (**A**) Time courses of cell growth (OD600) and glucose consumption during fermentation. Effects of fermentation duration, (**B**) inoculum size, (**C**) XAD-16 resin loading, (**D**) and amino acid precursor supplementation (**E**) on FK228 production. For panel D, compared with the resin-free control, 4% (w/v) XAD-16 resin increased the FK228 titer by approximately threefold. Data are represented as mean values ± SD (*n* = 3 biological independent samples).

Building on these physiological optimizations, we addressed substrate limitations by supplementing three key precursors-cysteine, valine and threonine-essential for FK228 assembly (Figure 1E). While valine alone gave a marginal 6% increase, combined feeding of all three precursors produced a synergistic effect, boosting the final titer to 743.5 mg/L (Figure 1E)-representing a 28% increase over the baseline titer of 581 mg/L. This result underscores that precursor availability may contribute to limiting the flux through the FK228 biosynthetic pathway under the tested conditions; however, direct metabolomic evidence would be required to establish precursor limitation as the sole or primary mechanism The biosynthetic logic and structural correlations are detailed in Supplementary Figure S3.

### 3.2 Specificity of transcriptional regulation in FK228 biosynthesis

Previous studies established that the AraC-family regulator TdpR and the LysR-family regulator DepR function as pathway-specific activators, each capable of enhancing the titers of their respective target product (Liu et al., 2018; Potharla et al., 2011). Although the BGCs for FK228 (*dep*) and thailandepsin (*tdp*) are highly conserved in gene content, organization, and amino acid sequence-sharing a common hybrid NRPS-PKS assembly line-their regulatory architechtures diverge strikingly. Specifically, *tdpR* encodes an AraC-type regulator, whereas *depR* encodes a LysR-type regulator, and the two proteins lack significant sequence homology (Wang et al., 2011). Given that pathway-specific regulators are critical for natural product biosynthesis, are relatively small in size, and are amenable to genetic manipulation, this regulatory dichotomy prompted us to investigate whether heterologous expression of the LysR-type regulator DepR could functionally replace the native AraC-type TdpR to sustain FK228 production in a heterologous host.

To test this hypothesis, we cloned *depR* from the *dep* BGC of *C. violaceum*strain No. 968 and inserted into the FK228 expression vector backbone, yielding the plasmid p15A-cm-depR-tdp-dep (Figure 2A). Redαβ-mediated recombination was subsequently employed to generate the conjugative plasmid p15A-km-int-*attP*-depR-tdp-dep (Figure 2B). Following conjugative transfer into the chassis strain KOGC1, the resulting engineered strain was designated KOGC1/depR-tdp-dep (Figure 2C). For comparative analysis, the previously constructed strain KOGC1/R-tdp-dep which expresses the native FK228 activator TdpR, was included as a positive control. Small-scale flask fermentation in M9 medium showed that KOGC1/depR-tdp-dep maintained detectable FK228 production at levels comparable to those of the control strain KOGC1/R-tdp-dep. This result demonstrates the ability of DepR to support FK228 biosynthesis in the heterologous KOGC1 background and suggests cross-pathway regulatory potential between these two regulators. However, whether DepR directly binds to and activates the FK228 BGC promoter or acts through an indirect, compensatory regulatory cascade remains to be determined (Figure 2D).

**Figure 2:**
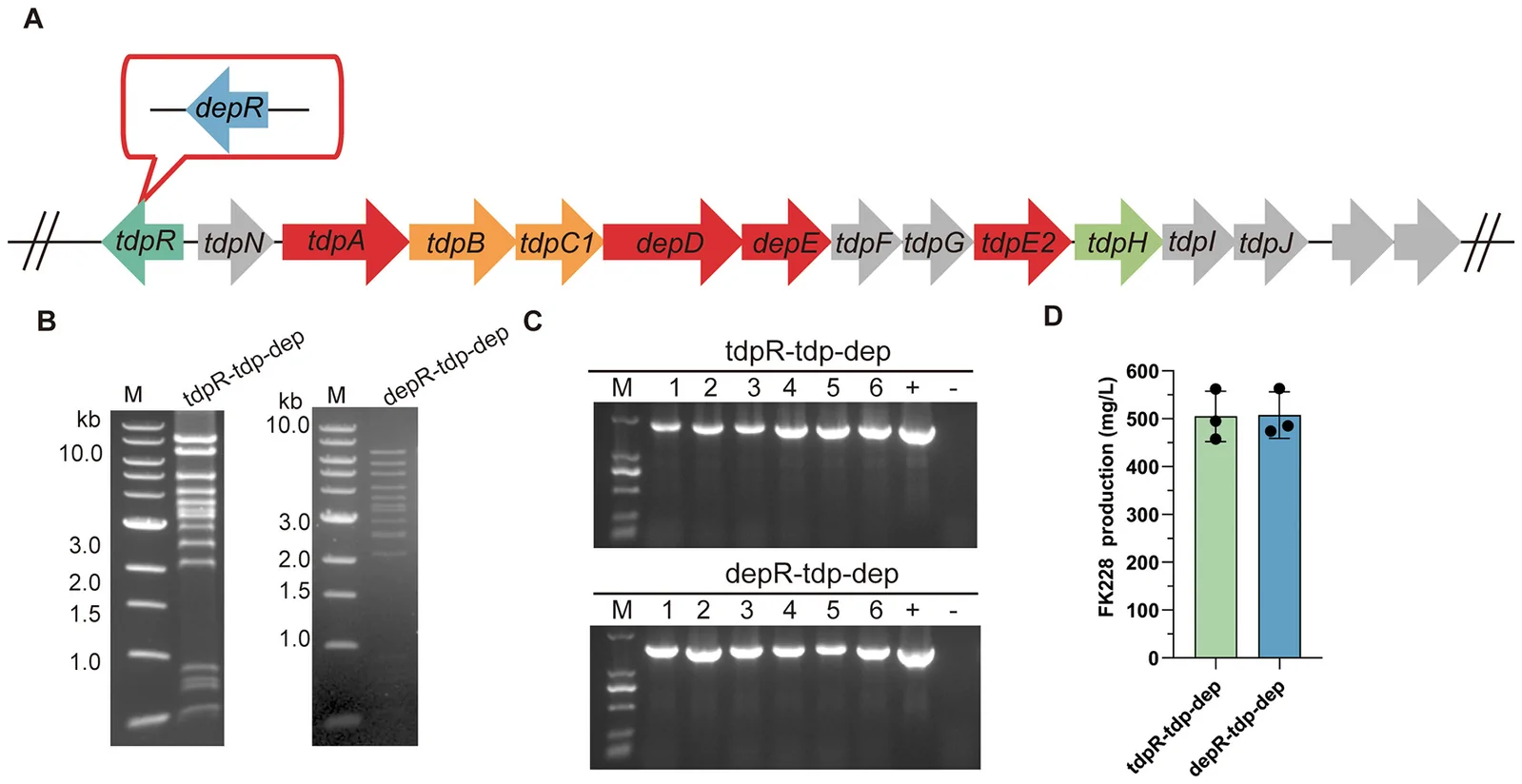
Transcriptional engineering of the FK228 biosynthetic pathway. **(A)** Schematic of the regulatory genes *depR* inserted into the FK228 expression vector. **(B)** Restriction analysis of the plasmids p15A-km-int-*attP*-R-tdp-dep and p15A-km-int-*attP*-depR-tdp-dep using *Pst*I digestion (M: 1 kb DNA ladder). **(C)** Conjugative transfer of the plasmids into the KOGC1 chassis strain. **(D)** FK228 production titers of the engineered strains. Data are represented as mean values ± SD (n = 3 biological independent samples).

Data are represented as mean values ± SD (*n* = 3 biological independent samples). *P* values were determined by two-tailed unpaired *t* test. ^*^p < 0.05; ^**^p < 0.01; ^***^p < 0.001.

### 3.3 Overexpression of TdpR to enhance FK228 production

Gene expression levels directly affect biosynthetic efficiency, with transcription activity largely governed by promoter strength. The AraC-type transcription factor TdpR has been identified as a positive regulator capable of enhancing the titers of thailandepsin. Consistent with this, our previous work demonstrated that inclusion of *tdpR* in the FK228 BGC carrying increased FK228 yield by 1.7-fold compared to the cluster without *tdpR*, confirming that TdpR promotes FK228 production through transcriptional activation of the BGC. Additionally, our group previously identified a series of highly efficient constitutive promoters from *B. thailandensis* E264, which enabled an increase in disorazol F2 titer to 38.3 mg/L (Ouyang et al., 2020; Wang et al., 2022).

To further enhance FK228 production, we used RedEx technology to insert seven strong constitutive promoters upstream of *tdpR* (Figure 3A and the detailed workflow is illustrated in Supplementary Figure S4). These promoters-P11 (putative protein promoter), P16 (DNA-binding protein HU-alpha), P17 (radical SAM protein promoter), P33 (putative protein promoter), P35 (peroxidase protein promoter), P44 (membrane protein promoter), and P46 (protein promoter containing the DUF4148 domain)-selected from a previously characterized library of 50 natural promoters in *B. thailandensis* E264 based on their exceptionally high transcriptional activities, as determined by *in vivo* luciferase reporter assays. There relative strenths ranked as P46 > P44 > P35 > P33 > P17 > P16 > P11 (Wang et al., 2022). To enable stable chromosomal integration, the chloramphenicol resistance cassette (*cmR*) was replaced with a *km-int-attP* integration module via Redαβ recombination (Figure 3B). The reassembled Px-tdpR-tdp-dep BGCs were then integrated into the strain KOGC1. Correct integration was verified by colony PCR, and the resulting strains were cultured in M9 medium using the optimized parameters (Supplementary Figure S5).

**Figure 3:**
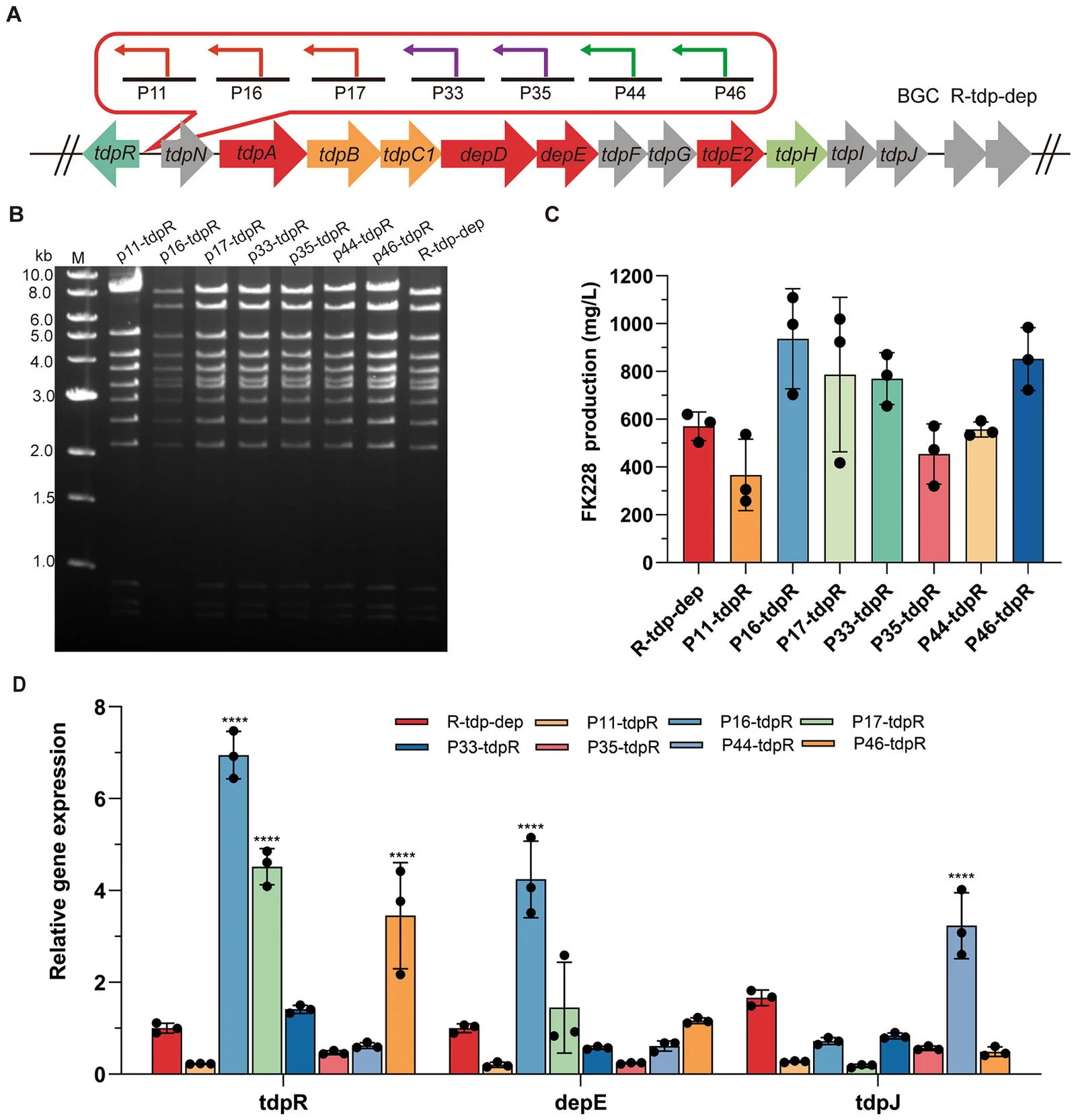
FK228 production by KOGC1 with different promoter strategies. **(A)** Overview of the construction of Px-tdpR-tdp-dep plasmids. **(B)** Restriction analysis of the plasmids p15A-km-int-*attP*-Px-tdpR-tdp-dep using *Pst*I digestion (M: 1 kb DNA ladder). **(C)** FK228 production titers of strain KOGC1 harboring Px-tdpR-tdp-dep BGC. **(D)** Relative transcript levels of *tdpR, depE*, and *tdpJ* in the seven promoter-engineered strains.Fermentation samples for RNA isolation were collected at 24 h. Relative transcription levels were normalized to *rpoB* and determined by the 2∧–ΔΔCt method. Values are shown as fold-changes versus KOGC1/R-tdp-dep. Error bars indicate standard deviations from three independent biological replicates (parallel independently inoculated cultures from individually picked single colonies; separate RNA extraction and reverse transcription per sample; two technical replicate wells averaged prior to analysis). Significant differences versus baseline are marked and Error bars indicate the standard deviations from three independent biological replicates, ****p < 0.0001.

HPLC-MS analysis demonstrated that overexpression of the pathway-specific activator TdpR significantly enhanced FK228 production. Among the promoters screened, P16 drove the highest titer, reaching 1109.4 mg/L (Figure 3C). This titer represents a 90% increase over the previously reported KOGC1 baseline of 581 mg/L and a 49% increase over the intermediate titer of 743.5 mg/L obtained through fermentation optimization alone. As summarized in Supplementary Table S5, this constitutes the highest FK228 titer reported to date, clearly demonstrating the synergistic effect of combining systematic process optimization with targeted promoter engineering. Other strong promoters also effectively drove *tdpR* expression, yielding titers of 870.2 mg/L (49.7 % increase) for P33, and 984.6 mg/L (69.4 % increase) for P46. Collectively, these results demonstrate that strategic promoter engineering of *tdpR* is a highly effective approach for maximizing the biosynthetic capacity of the heterologous FK228 pathway.

Finally, we performed qRT-PCR to assess transcriptional effects of promoter engineering on three FK228 biosynthetic genes (*tdpR, depE*, and *tdpJ*, using *rpoB* as an internal control. RNA was extracted from 24-h cultures of all seven engineered strain. Transcript levels of all three genes were elevated in strains P16, P17, and P46 relative to the parental control (Figure 3D), with P16 showing the highest mRNA levels. Notably, the chromosomal transcript ranking (P16 > P17 ≈ P46) did not macth the promoter-strength ranking derived from luciferase reporter assays (P46 > P44 > P35 > P33 > P17 > P16 > P11), indicating that surrogate reporter data poorly predict native in-locus transcription. Collectively, targeted *tdpR* promoter tuning enhanced BGC transcription, broadly correlating with titer increases, albeit with context-dependent divergence from the construct-design surrogate metrics.

### 3.4 Genetic stability of high-yielding FK228-engineered strains

To evaluate the genetic stability of the engineered high-yielding strains, three representative strains-KOGC1/R-tdp-dep, KOGC1/P16-tdpR-tdp-dep, and KOGC1/P46-tdpR-tdp-dep-were continuously passaged for five generations under non-selective, antibiotic-free conditions. Fermentation analysis revealed that all three strains maintained stable FK228 production across generations. Specifically, the titers of the first (G1), third (G3), and fifth (G5) generations of KOGC1/P16-tdpR-tdp-dep and KOGC1/P46-tdpR-tdp-dep were comparable to those of their respective parental strains, while KOGC1/R-tdp-dep similarly exhibited no significant titer fluctuation from G1 through G5 (Figures 4A, B, C). To verify the integrity of the integrated biosynthetic cassette, colony PCR was performed on four randomly picked colonies per strain at generations G1, G3, and G5, using primer pairs flanking the full refactored BGC. All amplicons matched the expected sizes, with no evidence of deletion, rearrangement, or cassette loss (Supplementary Figure S6). These results confirm short-term genotypic and phenotypic stability over five generations without antibiotic selection, although long-term stability under bioreactor or extended-culture conditions remains to be investigated.

**Figure 4:**
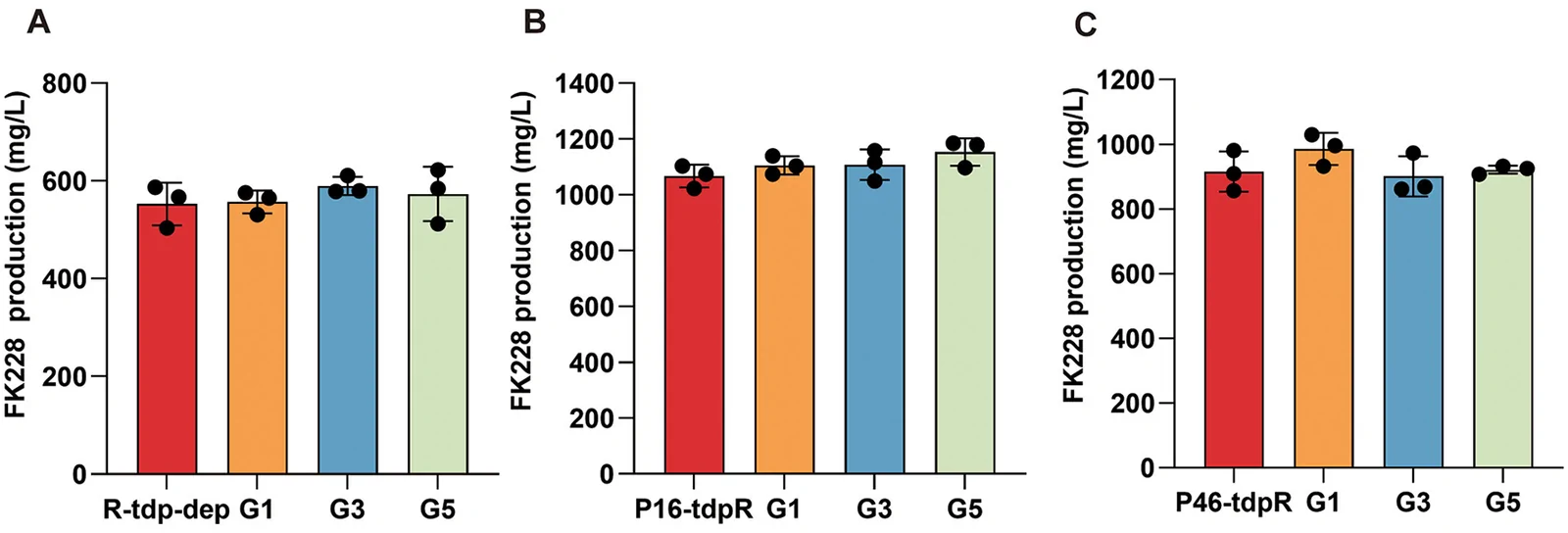
Genetic stability of three high-yielding FK228-engineered strains. Fermentation analysis of the first (G1), third (G3) and fifth (G5) generations of KOGC1/R-tdp-dep **(A)** KOGC1/P16-tdpR-tdp-dep **(B)** and KOGC1/P46-tdpR-tdp-dep **(C)** over five successive sub-cultivation in the absence of antibiotic selection. The fermentation samples were collected at 60 h. Data are represented as mean values ± SD (*n* = 3 biological independent samples).

## 4 Discussion

FK228, a potent antitumor natural product, has faced development constraints due to the genetic recalcitrance of its native producer. Although extensives studies on its mechanism and clinical use, bioproduction progress has remained limited. In this study, through optimized fermentation and promoter engineering, we achieved a titer of 1,109.4 mg/L-the highest reported titer for FK228 to date (Supplementary Table S5). Additionally, the engineered *Burkholderia* chassis maintained BGC integrity and stable productivity over five generations without antibiotic selection, a feature that addresses a critical bottleneck for industrial translation, establishes a robust platform bridging genetic potential with scalable manufacturing. It should be noted that the quantitative yield differences among engineered strains did not reach statistical significance under the current replication scheme; thus, titer variations are discussed as phenotypic trends. Nevertheless, the 1109.4 mg/L titer of the P16-engineered strain was consistently reproduced across multiple independent fermentation batches, supporting the robustness of this strain.

The comparable FK228 production driven by the heterologous LysR-type regulator DepR and the native AraC-type regulator TdpR suggests regulatory flexibility within the pathway's regulatory network. Although the *dep* and *tdp* biosynthetic gene clusters are highly conserved, the marked structural divergence between DepR and TdpR indicates that DepR may support FK228 biosynthesis through mechanisms distinct from those of TdpR, rather than acting as a direct functional analog. DepR sustains FK228 production in the heterologous host; however, whether DepR directly activates the FK228 BGC or functions through an indirect regulatory cascade remains unproven and is proposed here as a testable hypothesis. Future biochemical studies, such as EMSA and transcriptomic profiling, are needed to define the interaction between DepR and the *tdp* cluster and facilitate the design of synthetic transcriptional regulators.

The current study was conducted under laboratory-scale flask fermentation conditions (65 mL), and the engineered platform should therefore be evaluated in the context of its potential for future scale-up rather than immediate industrial applicability. Despite the laboratory-reported record-high titer, several challenges must be addressed before FK228 production can meet the requirements of large-scale manufacturing. First, the increased broth viscosity during the late fermentation stage, exacerbated by resin addition, currently hinders efficient downstream processing and extraction efficiency. Optimizing cell lysis protocols or developing continuous flow separation systems will be crucial to minimize product loss. Second, the metabolic burden imposed by the maintenance of large exogenous BGCs continues to threaten long-term strain stability. Although the engineered strain maintained genetic stability over five generations without antibiotic selection, stability beyond this period remains to be assessed; future efforts should focus on constructing more resilient *Burkholderia* chassis strains utilizing CRISPR-Cas-based genome stabilization techniques to prevent structural rearrangements or deletions. Lastly, the regulatory and biosynthetic mechanisms underlying the observed titer improvement remain incompletely characterized. While our results suggest that precursor supply and in-situ product adsorption may contribute to enhanced production, direct evidence for intracellular precursor limitation or end-product toxicity is lacking. Similarly, whether DepR directly activates the FK228 BGC or acts through an indirect regulatory cascade remains to be determined. Integrating systems biology approaches, such as multi-omics analysis, will help identify cryptic regulatory nodes or enzymatic inefficiencies. Addressing these multifaceted challenges through interdisciplinary engineering will pave the way for the future process development and sustainable production of FK228 and other high-value natural products.

## Data Availability

The original contributions presented in the study are included in the article/Supplementary material, further inquiries can be directed to the corresponding authors.
