# Supplementary material for: Engineering the FK228 biosynthetic gene clusters for high-yield production
Source: Front Microbiol. 2026 Oct 15;17:1933794. doi: 10.3389/fmicb.2026.1933794 (PMC13716795; doi:10.3389/fmicb.2026.1933794)
Supplement: Supplementary file 1 [file Supplementary_file_1.pdf]

Supplementary Material

Engineering the FK228 biosynthetic gene clusters for high-yield production

Maoqin Wang[1], Xuexin Jia[1], Yunling Zhang[1], Junchi Ma[1], Youming Zhang[1], Jun Fu[1,2,*], Ruijuan Li[1,*]

[1]State Key Laboratory of Microbial Technology, Shandong University, Qingdao 266237, China

[2]Shanghai Institute for Biomedical and Pharmaceutical Technologies, Shanghai, 200237, China

[*]Correspondence:

Jun Fu
fujun@sdu.edu.cn

Ruijuan Li
liruijuan@sdu.edu.cn

Contents

Table S1. Plasmids used in this study.

Table S2. Strains used in this study.

Table S3. Primers used in this study.

Table S4. Selected promoter sequence from *B. thailandensis* E264.

Table S5. Summary of reported FK228 production systems and titers.

Figure S1. Plasmid map of p15A-cm-R-tdp-dep.

Figure S2. Analytical validation of FK228 quantification and identity by HPLC-MS/MS

Figure S3. Biosynthetic pathway of FK228 and precursor incorporation strategy.

Figure S4. Seamless insertion of different promoter by RedEx strategies.

Figure S5. Colony PCR verification of overexpression of *tdpR* in KOGC1.

Figure S6. Colony PCR verification of cassette retention after serial passage.

Table S1. Strains used in this study.

Strains	Descriptions	Sources
Escherichia coli		
GB2005	(HS996, Δ*recET*, Δ*ybcC*). The endogenous *recET* locus and the DLP12 prophage *ybcC*, which encodes a putative exonuclease similar to the Redα, were deleted	Our lab
GBred-gyrA462	(GB05-red, *gyrA462*) GyrA mutation of Arg462Cys	Our lab
WM3064	*E. coli* donor strain for conjugation	Our lab
***Burkholderia thailandensis* E264**		
KOGC1	BGC *tdp* koockout*, with attB* site integrated	Gong et al. (our group)
KOGC1/R-tdp-dep	p15A-km-int-*attP*-tdpR-*tdp-dep* integrated to *attB* site of KOGC1	Gong et al. (our group)
KOGC1/P11-R-tdp-dep	p15A-km-int-*attP*-P11-R-*tdp-dep* integrated to *attB* site of KOGC1	This Work
KOGC1/P16-R-tdp-dep	p15A-km-int-*attP*-P16-R-*tdp-dep* integrated to *attB* site of KOGC1	This Work
KOGC1/P17-R-tdp-dep	p15A-km-int-*attP*-P17-R-*tdp-dep* integrated to *attB* site of KOGC1	This Work
KOGC1/P33-R-tdp-dep	p15A-km-int-*attP*-P33-R-*tdp-dep* integrated to *attB* site of KOGC1	This Work
KOGC1/P35-R-tdp-dep	p15A-km-int-*attP*-P35-R-*tdp-dep* integrated to *attB* site of KOGC1	This Work
KOGC1/P44-R-tdp-dep	p15A-km-int-*attP*-P44-R-*tdp-dep* integrated to *attB* site of KOGC1	This Work
KOGC/-P46-R-tdp-dep	p15A-km-int-*attP*-P46-R-*tdp-dep* integrated to *attB* site of KOGC1	This Work
KOGC1/depR-tdp-dep	p15A-km-int-*attP*-depR-*tdp-dep* integrated to *attB* site of KOGC1	This Work

Table S2. Plasmids used in this study.

Plasmids	Characteristics	Sources
p15A-cm-R-tdp-dep	p15A replicon, *kan*^{R}, *oriT-attP-phCi31* cassette, hybird t*dp-dep* BGC and *tdpR* regulatory gene	This work
p15A-km-int-*attP*-R-tdp-dep	p15A replicon, *kan*^{R}, *oriT-attP-phCi31* cassette, hybird t*dp-dep* BGC and *tdpR* regulatory gene	This work
p15A-km-int-*attP*-P11-tdpR-tdp-dep	p15A replicon, *kan*^{R}, *oriT-attP-phCi31* cassette, *tdpR* promoted by p11	This work
p15A-km-int-*attP*-P16-tdpR-tdp-dep	p15A replicon, *kan*^{R}, *oriT-attP-phCi31* cassette, *tdpR* promoted by p16	This work
p15A-km-int-*attP*-P17-tdpR-tdp-dep	p15A replicon, *kan*^{R}, *oriT-attP-phCi31* cassette, *tdpR* promoted by p17	This work
p15A-km-int-*attP*-P33-tdpR-tdp-dep	p15A replicon, *kan*^{R}, *oriT-attP-phCi31* cassette, *tdpR* promoted by p33	This work
p15A-km-int-*attP*-P35-tdpR-tdp-dep	p15A replicon, *kan*^{R}, *oriT-attP-phCi31* cassette, *tdpR* promoted by p35	This work
p15A-km-int-*attP*-P44-tdpR-tdp-dep	p15A replicon, *kan*^{R}, *oriT-attP-phCi31* cassette, *tdpR* promoted by p44	This work
p15A-km-int-*attP*-P46-tdpR-tdp-dep	p15A replicon, *kan*^{R}, *oriT-attP-phCi31* cassette, *tdpR* promoted by p46	This work
p15A-km-int-*attP*-depR-tdp-dep	p15A replicon, *kan*^{R}, *oriT-attP-phCi31* cassette, hybird t*dp-dep* BGC and *depR* regulatory gene	This work

Table S3. Primers used in this study.

Primers	Sequence (5'-3')	Applications
kmccdB for tdpR-1	GAGCGGAGTCACTCTCGCATGGTACCCCGGAATTGCCAG	p15A-km-ccdB-Px-R-tdp-dep construction
kmccdB for tdpR-2	CCACATGGCTTCACTATCAAGACCGCGATCATGCGAGAGTGACTCCGCTCGGTACCTTTGTTCAAAAAA	
P11-tdpR-1	GTCTCGGACAATACTTCGCACCGGTACATGCCGCATACGTCGGTCTGCATGATTCCTCCTTCGTCGTAGTC	p15A-km-ccdB-P11-R-tdp-dep construction
P11-tdpR-1	ATGCGAGAGTGACTCCGCTCTTGACGGCCCGAGGCGCGTCT	
P16-tdpR-1	GTCTCGGACAATACTTCGCACCGGTACATGCCGCATACGTCGGTCTGCATTAAGGTTCCTTTCCAGGTTAG	p15A-km-ccdB-P16-R-tdp-dep construction
P16-tdpR-2	ATGCGAGAGTGACTCCGCTCGCGAAACAAAAAAGAAGGCGG	
P17-tdpR-1	GTCTCGGACAATACTTCGCACCGGTACATGCCGCATACGTCGGTCTGCATGCGCATTCCCCTCGATATGGG	p15A-km-ccdB-P17-R-tdp-dep construction
P17-tdpR-2	ATGCGAGAGTGACTCCGCTCCGTGTAATGCCTGAAACCGTC	
P33-tdpR-1	GTCTCGGACAATACTTCGCACCGGTACATGCCGCATACGTCGGTCTGCATCAGCATTCTCCCGGCGGGGAC	p15A-km-ccdB-P33-R-tdp-dep construction
P33-tdpR-2	ATGCGAGAGTGACTCCGCTCCGTGTAATGCCTGAAACCGTC	
P35-tdpR-1	GTCTCGGACAATACTTCGCACCGGTACATGCCGCATACGTCGGTCTGCATGTTGCAGCTCCTTGAACTTGC	p15A-km-ccdB-P35-R-tdp-dep construction
P35-tdpR-2	ATGCGAGAGTGACTCCGCTCTGCGACTCCCTTCTTGCCGAG	
P44-tdpR-1	GTCTCGGACAATACTTCGCACCGGTACATGCCGCATACGTCGGTCTGCATATTTCTCCTCTCGAAATTGAG	p15A-km-ccdB-P44-R-tdp-dep construction
P44-tdpR-2	ATGCGAGAGTGACTCCGCTCGCGGCAAGCGGTGTTGCACTC	
P46-tdpR-1	GTCTCGGACAATACTTCGCACCGGTACATGCCGCATACGTCGGTCTGCATGATTGACCTCCGGAATGTTGT	p15A-km-ccdB-P44-R-tdp-dep construction
P46-tdpR-2	ATGCGAGAGTGACTCCGCTCCCGGCTTTCGGTCGAAATTAA	
tdpR-seq-check	CATGTGGGCGCATCGGCTGA	p15A-km-ccdB-Px-R-tdp-dep check
km-int-attP-1	ACTAGTGCTTGGATTCTCACCAATAAAAAACGCCCCATGCTACGTAAATTCACTGGCCG	*kan*^{R}, *oriT-attP-phCi31* cassette PCR
km-int-attP-2	CGTATATCGAAACACAGTCGTCACAATTCAGCTGACTGCATCCGATGCAAGTGTGTCG	
Px-check-1	CCTGCGCGATCGCGAGCATCGTGTC	the verification of colony PCR
Px-check-2	TCAACCTTAACATAGAAAATAA	
kmccdB for regulator-1	GAACCTCTTACGTGCCGATCAACGTCTCATTTTCGCCAGATATCGACGTCGGTACCCCGGAATTGCCAGCTGGGG	p15A-km-ccdB-depR-tdp-dep construction

kmccdB for regulator-2	GACGTCGATATCTGGCGAAAGGTACCTTTGTTCAAAAAAAAGCCC	
depR-PCR-1	TTTCGCCAGATATCGACGTCTCAGGGCGTTGGCGTTGCGC	
depR-PCR-2	AAGCGCCTTCGCTCACCGCCCTCGAATCAGGCCGCATAGCACTGGATCATATGACGCTG ACCGAACTTCGCTA	p15A-km-ccdB-depR-tdp-dep construction
km-int-attP-DepR-2	CACCCTGCAACAGGCGGTGTTCCGCTCCGGGCTGGAAGGCGTTTCCATGCTGGAAGGCG CAACGCCAACGCCCTGACTGCATCCGATGCAAGTGTGTCG	km-*int*-attP for depR cassette
DepR-check-1	ACGAAGTCGACTACGGGCAGG	p15A-km-ccdB-depR -tdp-dep check
Regulator-Colony-2	CACATCAGTGCACACGCATTGC	
depDE-ColonyPCR-1	CCGCCGCGTTCGAACTGGCCCAACA	
depDE-ColonyPCR-1	TCTGCTGCTGGATGGTTGGTCGGT	the verification of FK228 BGC colony PCR
tdpA-ColonyPCR-1	GGCTCATCGACCTCGGACTCGATTC	
tdpA-ColonyPCR-1	CCGCACGCGTATTGCAGGTCACGTT	
BGC-F-check-1	CGCCCGCGCTTTCCGCACTGC	
BGC-F-check-2	GTTACGCCGGCGGTAGCCGGC	Colony PCR, G1/G3/G5 BGC-verification
BGC-R-check-1	GATGCGCCGATACGAGAGGTGCT	
BGC-R-check-2	TGCTTGCGTCCGCCTTGCCGCG	
rpoB-check-1	CGAAGCGTTCCATCGTTCAC	
rpoB-check-2	GCACATCGGCTTGCAGAAAT	
tdpR-check-1	CGCCCACATGGCTTCACTAT	
tdpR-check-2	TGGCGATTGCTCTCGATCTC	qPCR analysis
depE-check-1	CGTCGGACGTATGGACCTTGT	
depE-check-2	CAGCGGTAAGGCACGATTTCC	
tdpJ-check-1	TTCTGGACGAGGTGATACGC	
tdpJ-check-2	GCCTTGAAATCGGCACGAAG	

* The underlined bases represent homologous arms (HAs), the double underlined bases represent single restriction enzyme site

Table S4. Selected promoter sequence from *B. thailandensis* E264.

**Promoter**	**Gene ID**	**Sequence (5'-3')**
P11	BTH_RS10190	ttgacggcccgaggcgcgtctatatgatgaattggccgacaaacgggcggaccgcaccgctcggcccgcgcctgcactccaaaccaccgcacgactacgacgaaggaggaatc
P16	BTH_RS12210	gcgaaacaaaaaagaaggcggcgaaaatgcggcgaaccgccgcgaaagccccgtccagcctggcgttggcaaaatccaacgaaaccgcgcgcgggcgttggccgagccgcggccaagccggct<br>cgcgcgactcgaaagccgcgcggaacgcgtctaactcgacaaaattcgctcgaaaatctttgaaacccttgtcgttattgggtttgcgctgcgctatcatagcaagcgccgtatcgcggagctgccaggag<br>gcaaacccggcggtgcgttgcgcggcgcaatactttgcgccgtgccgttgccacacactatagacggcacggttcgcgcacgtataatcggcgcgttcgcgctgttcgtgtcaacctaacctggaaagga<br>acctta
P17	BTH_RS08720	cgtgtaatgcctgaaaccgtcaggtggtaatgtcgacgagtcgcgtcgagcgcggcatcccacaccaggcgataaacatgcagtgtgcgcttctcgcatggaatcatgcgcacgactccgacgcggtgg<br>tcgacgaggacgtcctgccccacaaccgtcgggcactcaggcgggaaagaacgtcccaactcgcataagcgttcgcagcgtgacgccgggagcggtctcgagcacaccacaaagcccaagtgcgca<br>tgcgtgccgccatgaatcgccaccgtacgcgccgggatgcgccgtcgcatcgagtttgaagggggagtccaaaaatcgttcatgacggctcatcgtctttgtgattgcgcatcgcagccattcatccatcca<br>agccggcgtaagagactttacccgggtcactgttccttgactttcagcatatatcatccattattaggctcgttgcccatatcgaggggaatgcgc
P33	BTH_RS00265	gcgctcggccccgttgtgatccgatccggtaaagcgtacgctgttcgttccgacgtcggttcgcctcccccgcgaatccgtccccgccgggagaatgctg
P35	BTH_RS19270	tgcgactcccttcttgccgaggcgggttgtgctacggacatcgtagcgaaacccgtgcctgcgcgcgggtgcggcggcacggcacgcaggcagcgcaagatcgcgcttgagtagcatgatggtaaac<br>gacttgtcgagacaatagagagccgttccggaacgaaaaagtttccaggattgtcaaatcgcacccggcgaaaatgataaaaaggcatgaatatctgacggaataatcgttcgcgccggccgcgggatca<br>cttaagcttaaagcttgtccggatgcgttctgtattcgggcgcccaacccatgcaagttcaaggagctgcaac
P44	BTH_RS20520	gcggcaagcggtgttgcactcgcgccacaatcgatagaccgatgttagggtggggaggattttttttcgtgggaagggaacgatatggcaaaatgccaacgcactgaaagttagacgctgctcgaagtcta<br>cacagagcggtgccgcgttttttgtgccgcaccaatgttatacttcgagcaatgtatgacttgtcatcgtcaacaggctcgcagtaaacctgcggtaatctcaatttcgagaggagaaat
P46	BTH_RS07755	ccggctttcggtcgaaattaaccggctttcggtcgaaatacagtccgccatttttatcattggcgcaacgctgtatgtctaaagtaggggtttccccttgctgttccggtgactagactggaatcaatcgcttccg<br>gccaggatgcctggagcgacgcaaatacaacattccggaggtcaatc

Table S5. Summary of reported FK228 production systems and titers

**Host strain**	**Engineering strategy**	**Medium/Scale**	**FK228 titer (mg/L)**	**Reference**
C. violaceum No.968(native)	None (pilot-scale batch)	Industrial piolot batch	~19	Liu et al.
B. thailandensis MSMB43(native)	None	M8, flask	67.7	Liu et al.
B. thailandensis MSMB43(native)	None	M8, 20-L fed-batch	115.9	Liu et al.
B. thailandensis MSMB43	Plasmid-borne tdpR overexpression (pBMTL3-tdpR)	M8, 20-L fed-batch	168.5	Liu et al.
E. coli BL21 (heterologous)	Chemically synthesized dep BGC	Flask	~19	Cheng et al.
E. coli Nissle 1917 (heterologous)	Codon-optimized dep BGC,promoter refactoring	Flask (*in vitro*)	1.46-1.5	Ma et al.
KOGC1	Hybrid R-tdp-dep BGC, phiC31 integration	M9, flask, 60h	581	Gong et al. (Our group)
KOGC1 (fermentation optimized)	Hybrid R-tdp-dep BGC, XAD-16 resin	M9, flask, 60h	~650	This study
KOGC1 (fermentation optimized)	Hybrid R-tdp-dep BGC, XAD-16 resin, precursor feeding	M9, flask, 60h	743.5	This study
KOGC1/P16-tdpR	Hybrid R-tdp-dep BGC, P16 promoter on tdpR	M9, flask, 60h	1109.4	This study

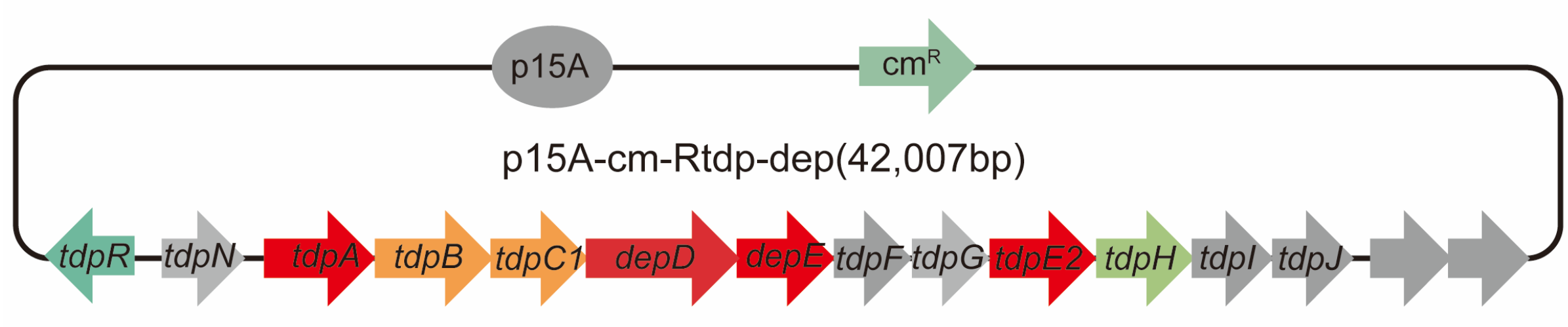

Figure S1. Schematic diagram of the FK228 biosynthetic plasmid p15A-cm-R-tdp-dep and its derivatives. The plasmid is based on a p15A origin of replication and carries a chloramphenicol resistance gene (cmR) for selection. The core FK228 biosynthetic gene cluster (tdp-dep) is driven by native or engineered promoters.

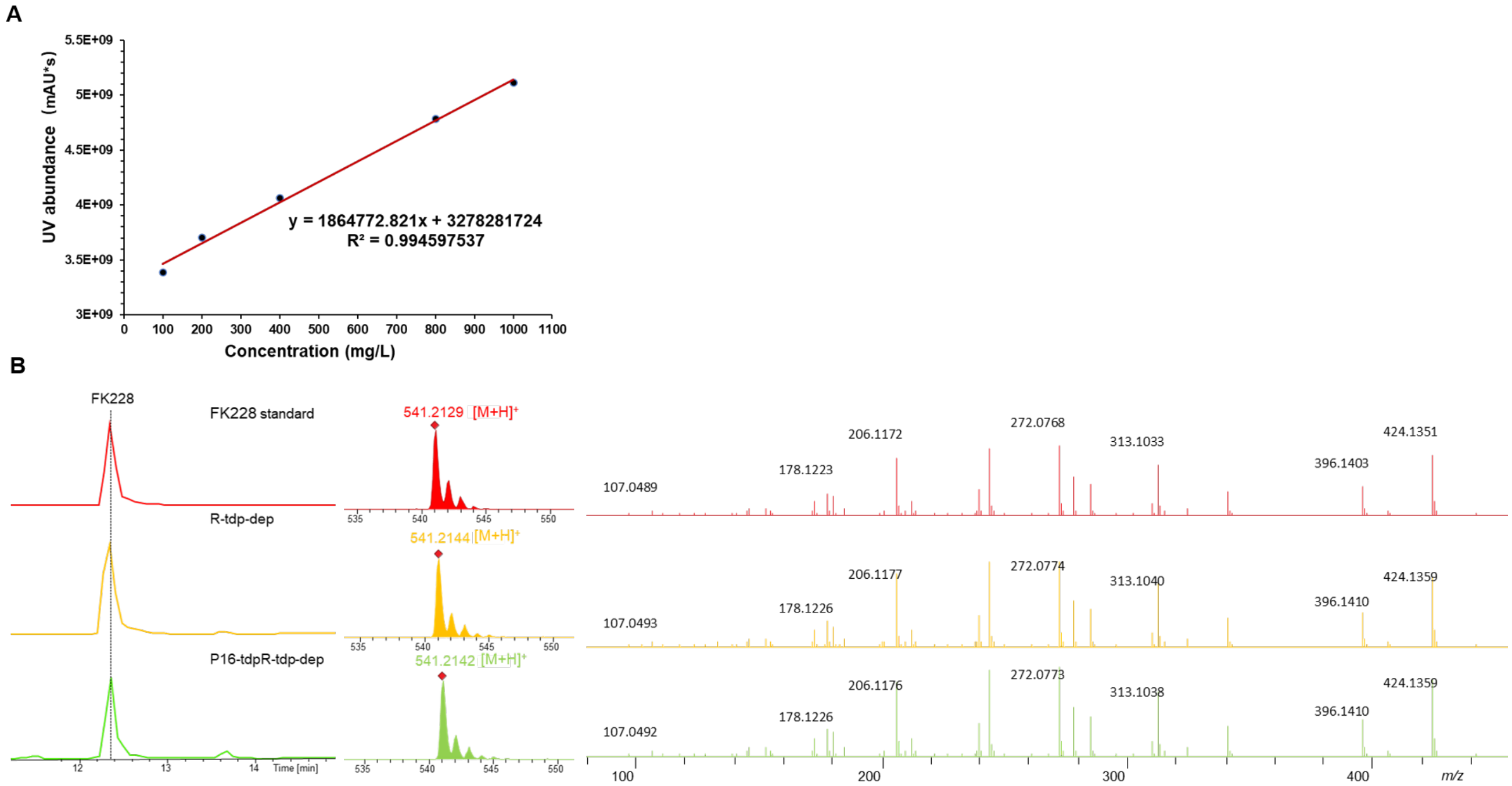

Figure S2. Analytical validation of FK228 quantification and identity by HPLC-MS/MS **(A)** Quantitative analysis of FK228 by HPLC-MS. Corresponding standard curve plotting peak area against concentration. The equation of the linear fit is $y=1864772.821x+3278281724(R^2=0.994)$, demonstrating high sensitivity and accuracy for FK228 quantification in the fermentation broth. **(B)** The FK228 chromatographic peaks were identified using HPLC-MS/MS. Extract ion chromatograms (EICs) of the FK228 standard (red curve) and representative fermentation samples (R-tdp-dep, yellow curve; P16-tdpR-tdp-dep, green curve) showed consistent retention times of 12.3 min. Comparison of MS/MS fragmentation patterns: FK228 standard (top) and the target peak in the fermentation extract (bottom). The matching fragmentation patterns confirm that this quantitative peak corresponds specifically to FK228, rather than structurally related metabolites.

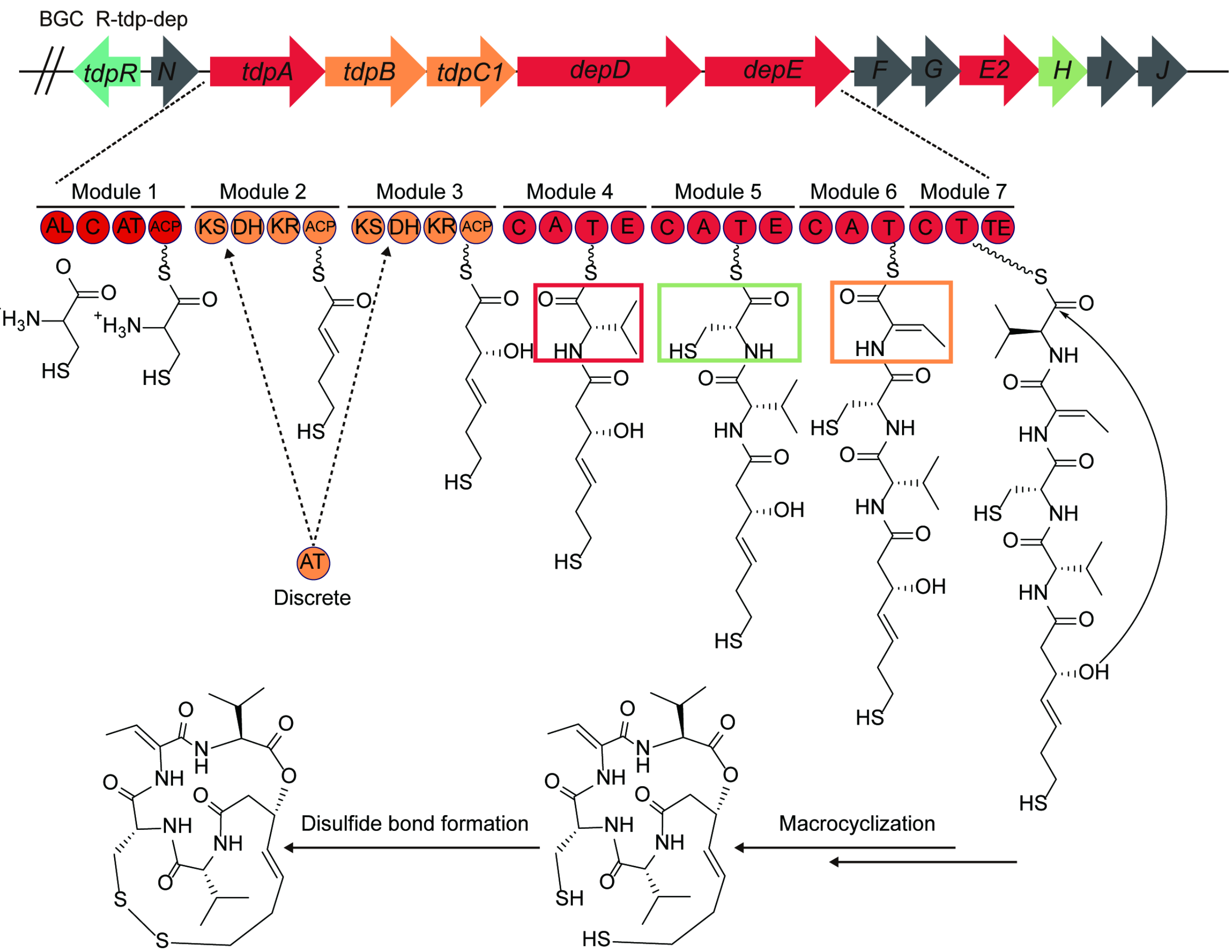

Figure S3. Biosynthetic pathway of FK228 and precursor incorporation strategy. Schematic representation of the FK228 (romidepsin) biosynthetic gene cluster (tdp) and the hybrid NRPS-PKS assembly line. Key domains are indicated.Chemical structure of FK228 highlighting the origin of specific subunits derived from exogenous precursors. The moieties derived from cysteine, valine and dehydrothreonine are highlighted in red, green, and yellow, respectively.

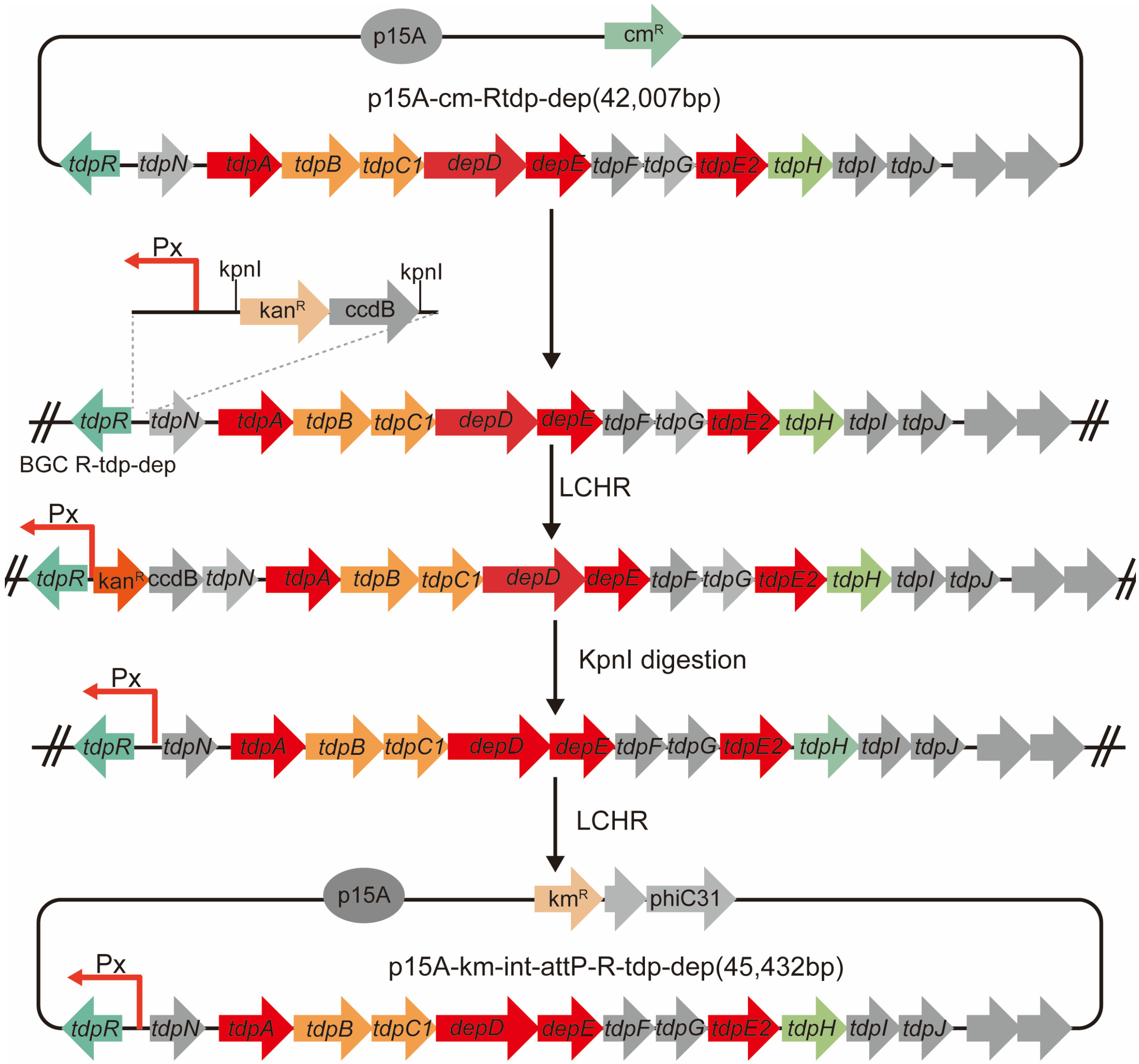

Figure S4. Promoter engineering of the *tdpR* using the RedEx strategy.The workflow comprises three main steps: (i) Insertion of a *kmccdB* counter-selection cassette upstream of *tdpR* in the plasmid p15A-cm-R-tdp-dep via LCHR. (ii) Removal of the counter-selection marker by KpnI digestion and subsequent Gibson assembly with a promoter library (P11–P46) to generate p15A-cm-Px-R-tdp-dep. (iii) Insertion of the km-int-*attP* site-specific recombination module. The km-int-attP integration cassette, containing the integrase gene (*int*) and the *attP*attachment site, was introduced via LCHR. This enables permanent integration of the reconstructed gene cluster into the *attB*locus of the KOGC1 chromosome via Int-mediated site-specific recombination.

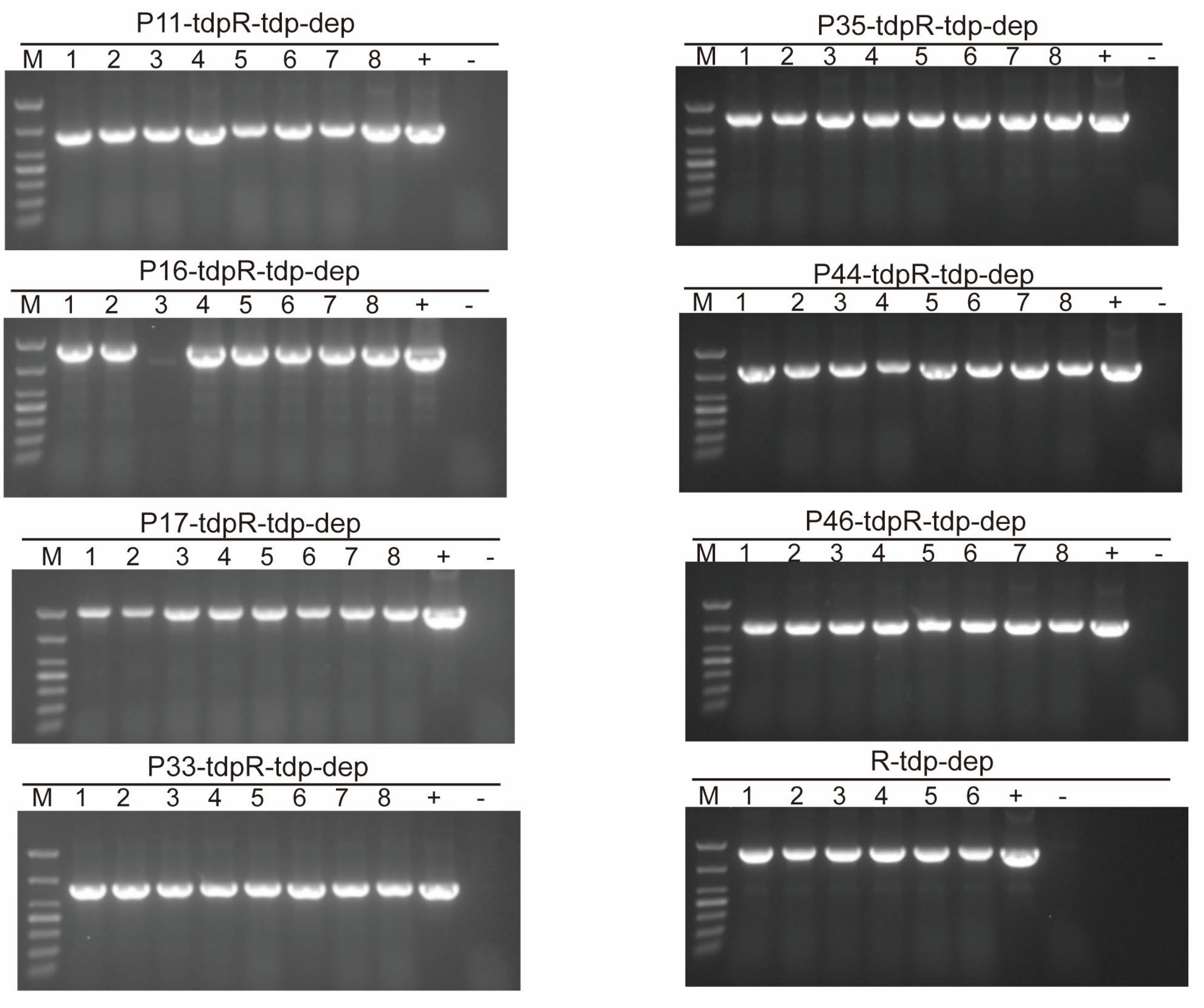

Figure S5. Molecular verification of the engineered KOGC1 strains.Colony PCR was performed to confirm the successful integration of the promoter-engineered FK228 gene cluster. Agarose gel electrophoresis of the PCR products. Lane M: 1000 DNA ladder. Lanes 1–8 correspond to strains integrated with promoters P11 through P46. (+ Positive control, - negative control)

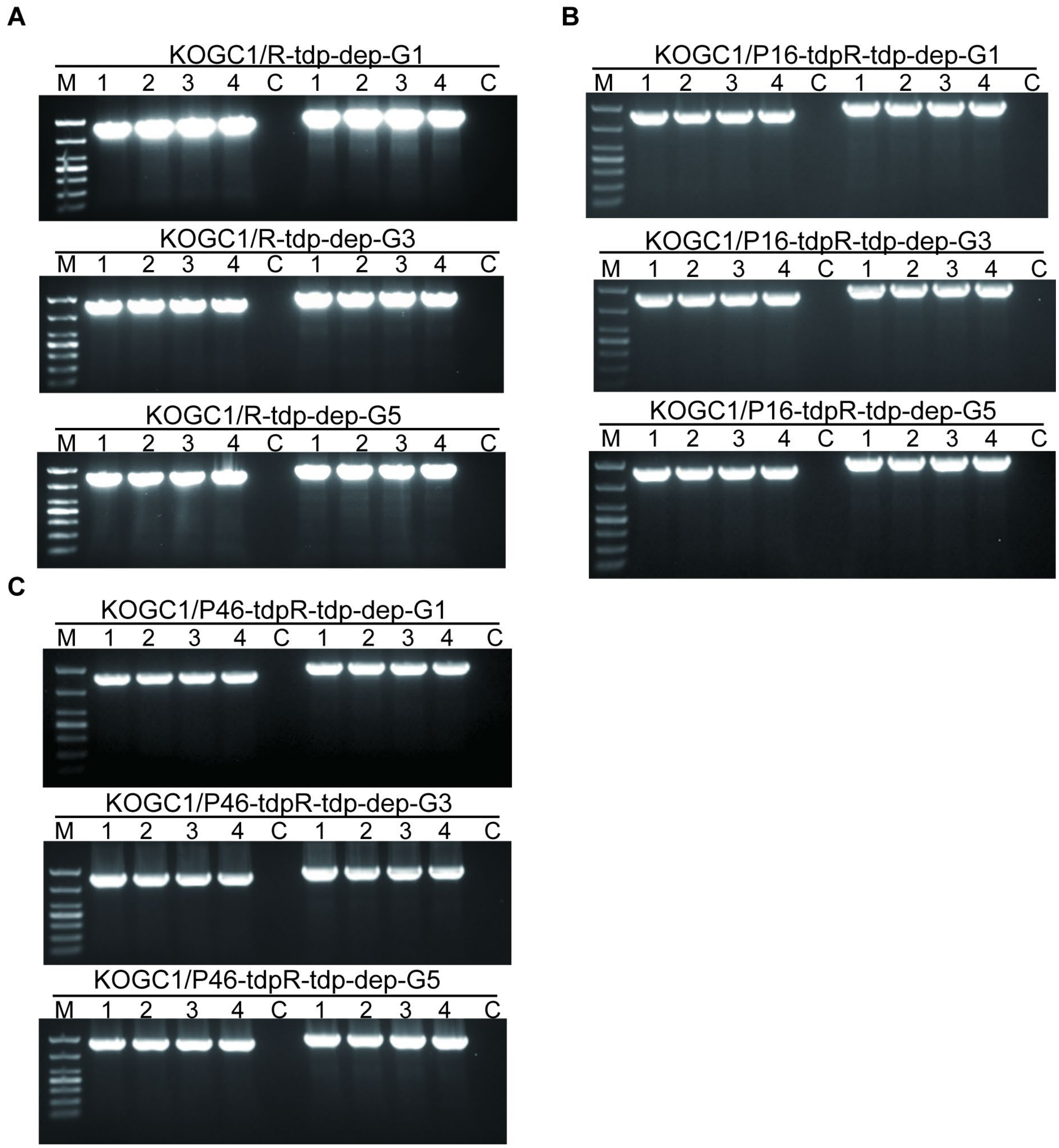

Figure S6. Colony PCR verification of cassette retention after serial passage. Four randomly picked colonies per strain at G1 **(A),** G3 **(B)**, and G5 **(C)** were screened with two primer pairs targeting the 5′ and 3′ integration boundaries of the refactored FK228 BGC. All amplicons matched the expected fragment sizes (5′ junction,861bp; 3′ junction, 975 bp) with no aberrant, truncated, or absent bands, indicating retention of the integrated cassette without detectable deletion or rearrangement over five generations of non-selective culture. Lanes: M, DNA 1000bp ladder; strain and generation labels as indicated. KOGC1/R-tdp-dep, KOGC1/P16-tdpR-tdp-dep, and KOGC1/P46-tdpR-tdp-dep as in panels A-C.

References

Wang, Z.-J., Liu X., Zhou H., Liu Y., Zhong L., Wang X., et al. Engineering of *Burkholderia thailandensis* strain E264 serves as a chassis for expression of complex specialized metabolites. Frontiers in Microbiology. 2022;13:1073243 http://doi.org/10.3389/FMICB.2022.1073243

Song, C., Luan J., Li R., Jiang C., Hou Y., Cui Q., et al. RedEx: a method for seamless DNA insertion and deletion in large multimodular polyketide synthase gene clusters. Nucleic Acids Research. 2020;48:e130–e30 https://doi.org/10.1093/nar/gkaa956

Gong, K., Wang M., Duan Q., Li G., Yong D., Ren C., et al. High-yield production of FK228 and new derivatives in a *Burkholderia* chassis. Metabolic Engineering. 2023;75:131–42 https://doi.org/10.1016/j.ymben.2022.12.002

Liu, X., Xie F., Doughty L. B., Wang Q., Zhang L., Liu X., et al. Genomics-guided discovery of a new and significantly better source of anticancer natural drug FK228. Synthetic and Systems Biotechnology. 2018;3:268–74 https://doi.org/10.1016/j.synbio.2018.10.011

Wang C., Wesener SR., Zhang H., Cheng YQ. An FAD-dependent pyridine nucleotide-disulfide oxidoreductase is involved in disulfide bond formation in FK228 anticancer depsipeptide. Chem Biol. 2009;16(6):585-593. https://doi: 10.1016/j.chembiol.2009.05.005

Ma C., Li G., Sun T., Tang X., Qiu T., Song J., et al. Engineered romidepsin biosynthetic pathways in Escherichia coli Nissle 1917 improve the efficacy of bacteria-mediated cancer therapy. PLoS Biol. 2026;24(3):e3003657. https://doi: 10.1371/journal.pbio.3003657
